# Unilateral Ureteral Obstruction for 28 Days in Rats Is Not Associated with Changes in Cardiac Function or Alterations in Mitochondrial Function

**DOI:** 10.3390/biology10070671

**Published:** 2021-07-16

**Authors:** Rodrigo Prieto-Carrasco, Alejandro Silva-Palacios, Pedro Rojas-Morales, Omar Emiliano Aparicio-Trejo, Estefany Ingrid Medina-Reyes, Estefani Yaquelin Hernández-Cruz, Carlos Sánchez-Garibay, Citlaltepetl Salinas-Lara, Natalia Pavón, Francisco Javier Roldán, Cecilia Zazueta, Edilia Tapia, José Pedraza-Chaverri

**Affiliations:** 1 Department of Biology, Faculty of Chemistry, National Autonomous University of Mexico (UNAM), Mexico City 04510, Mexico; rodrigo_prieto@my.uvm.edu.mx (R.P.-C.); pedrorojasm@comunidad.unam.mx (P.R.-M.); svarta02@comunidad.unam.mx (O.E.A.-T.); medinaingrid0@gmail.com (E.I.M.-R.); estefani.hernandez@quimica.unam.mx (E.Y.H.-C.); 2 Department of Cardio-Renal Pathophysiology, National Institute of Cardiology Ignacio Chávez, Mexico City 14080, Mexico; edilia.tapia@cardiologia.org.mx; 3 Department of Cardiovascular Biomedicine, National Institute of Cardiology Ignacio Chávez, Mexico City 14080, Mexico; alejandro.silva@cardiologia.org.mx (A.S.-P.); ana.zazueta@cardiologia.org.mx (C.Z.); 4 Department of Neuropathology, National Institute of Neurology and Neurosurgery Manuel Velasco Suárez, Mexico City 14269, Mexico; carlossanchez@innn.edu.mx (C.S.-G.); cisala69@hotmail.com (C.S.-L.); 5 Department of Pharmacology, National Institute of Cardiology Ignacio Chávez, Mexico City 14080, Mexico; natalia.pavon@cardiologia.org.mx; 6 Department of External Consultation, National Institute of Cardiology Ignacio Chávez, Mexico City 14080, Mexico; roldan@cardiologia.org.mx

**Keywords:** inflammation, renal damage, UUO, cardiac function, mitochondria

## Abstract

**Simple Summary:**

This work shows that renal damage produced by the demonstration of unilateral ureteral obstruction (UUO) is not related to cardiac dysfunction or the change in mitochondrial bioenergetics parameters, despite the inflammatory state.

**Abstract:**

Our work evaluated cardiac function and mitochondrial bioenergetics parameters in hearts from male Wistar rats subjected to the UUO model during 28 days of progression. We measured markers of kidney damage and inflammation in plasma and renal fibrosis by histological analysis and Western blot. Cardiac function was evaluated by echocardiography and proteins involved in cardiac damage by Western blot. Oxygen consumption and transmembrane potential were monitored in cardiac mitochondria using high-resolution respirometry. We also determined the activity of ATP synthase and antioxidant enzymes such as glutathione peroxidase, glutathione reductase, and catalase. Our results show that, although renal dysfunction is established in animals subjected to ureteral obstruction, cardiac function is maintained along with mitochondrial function and antioxidant enzymes activity after 28 days of injury evolution. Our results suggest that renocardiac syndrome might develop but belatedly in obstruction-induced renal damage, opening the opportunity for treatment to prevent this condition.

## 1. Introduction

Ureteral obstruction is a common clinical disorder that can ultimately cause renal injury [1]. The obstructed kidney has reduced renal blood flow and glomerular filtration rate, hydronephrosis, interstitial inflammatory infiltration, and tubular cell death. Progression to the hydronephrotic kidney is characterized by a marked loss of renal parenchyma and severe fibrosis observed after 1–2 weeks [2]. These events are closely related to molecular processes such as apoptosis, oxidative stress, and inflammation, which together lead to progressive tubulointerstitial fibrosis [3]. Unilateral ureteral obstruction (UUO) is a valuable model to elucidate both the pathogenesis and the mechanisms responsible for progressive renal fibrosis [2]. Significantly, this model resembles obstructive nephropathy in patients. For example, acute and chronic urinary obstruction due to benign prostatic hypertrophy or renal calculi is frequently reported in the clinic setting. In addition, a significant cause of renal failure in children and infants is related to congenital obstructive nephropathy [4,5].

Recent data show that plasma levels of tumor necrosis factor-alpha (TNF-α), interleukin-6 (IL-6), and IL-10 increased significantly with a maximum peak at 28 days in the UUO model [6]. Furthermore, macrophage infiltration and increased cytokine production and the up-regulation of TNF-α promote severe renal inflammation and fibrosis [7], indicating that inflammation plays a pivotal role in obstruction-induced renal damage.

The kidneys are the second most energy-demanding organ, behind only the heart [8]. The above is very relevant as acute kidney injury (AKI) is associated with increased heart failure and mortality [6]. Paradoxically, renal obstruction and its effects on heart function are not fully described. In this regard, pathological cardiac remodeling has been documented to occur 21 days after UUO in mice [9]. In contrast, Prud’homme et al. [6] reported that ureteral obstruction and the associated systemic inflammatory response induce cardiac fibrosis, left ventricle (LV) dilatation, and decreased shortening in mice subjected to renal obstruction. It has been suggested that the inflammatory response might impact other tissues, such as the heart.

Mitochondria play a fundamental role in establishing and progressing chronic kidney disease (CKD) [10,11]. However, in UUO-induced obstructive nephropathy, the mitochondrial function has not been thoroughly evaluated. Martínez-Klimova et al. [12] reviewed some features of mitochondrial function on UUO-induced obstructive nephropathy, whereas Bianco et al. [13] reported that after 14 days of UUO in mice, the contralateral kidney exhibits impaired mitochondrial function and altered redox homeostasis.

Therefore, under the hypothesis that the inflammatory response derived from the obstructed kidney [6] could affect other tissues such as the heart, we evaluated cardiac function and mitochondrial bioenergetics in male Wistar rats subjected to UUO during 28 days of evolution.

## 2. Materials and Methods

### 2.1. Reagents

The reagents used in this study were adenosine diphosphate (ADP) sodium salt, antimycin A, bovine serum albumin (BSA), fatty acid (FA)-free BSA, carbonyl cyanide m-chlorophenylhydrazone (CCCP), D-mannitol, eosin, ethylenediaminetetraacetic acid disodium salt dihydrate (EDTA), ethylene glycol-bis(2-aminoethyl ether)-N,N,N′,N′-tetraacetic acid (EGTA), 4-(2-hydroxyethyl)-1-piperazineethanesulfonic acid (HEPES), hematoxylin, horseradish peroxidase (HRP), K-lactobionate, magnesium chloride (MgCl_2_), nicotinamide adenine dinucleotide 2′-phosphate-reduced (NADPH), nicotinamide adenine dinucleotide phosphate (NADP^+^), oligomycin, phenylmethylsulfonyl fluoride (PMSF), rotenone, safranin O, Sirius red, sodium chloride (NaCl), sodium dodecyl sulfate (SDS), sodium glutamate, sodium malate, sodium phosphate monobasic (NaH_2_PO_4_), sodium pyruvate, sodium succinate dibasic, sucrose, taurine, and tris hydrochloride (Tris-HCl), which were purchased from Sigma-Aldrich (St. Louis, MO, USA). Sodium pentobarbital (PISABENTAL^®^, Mexico City, Mexico), used as a sedative and anesthetic, was purchased from Proveedora Veterinaria Kan S. A. de C. V. (Mexico City, Mexico).

### 2.2. Animals and Ethical Guidelines

Adult male Wistar rats (250–300 g) were fed a regular rodent chow diet (Laboratory rodent diet 5001; PMI Feed Inc., Richmond, IN, USA) with free access to water. They were housed in a temperature-controlled environment with a 12:12 h light–dark cycle until the beginning and end of the experiment. All procedures were approved by the Institutional Animal Care Committee (Comité Institucional para el Cuidado y Uso de Animales de Laboratorio, CICUAL) at the Faculty of Chemistry (FQ/CICUAL/260/18). In addition, experiments were performed according to the Mexican Official Guidelines for the Production, Use, and Care of Laboratory Animals (NOM-062-ZOO-1999), the Guidelines for the Disposal of Biological Residues (NOM-087-SEMARNAT-SSA1-2002), and in compliance with ARRIVE guidelines [14].

### 2.3. Antibodies

The antibody against fibronectin (FN, F3648) was purchased from Sigma-Aldrich (St. Louis, MO, USA). The antibodies against glyceraldehyde-3-phosphate dehydrogenase (GAPDH, G8795) were purchased from Sigma-Aldrich and Santa Cruz Biotechnology (sc-47724, Dallas, TX, USA). The antibody for cardiac troponin-T (TnT, ab8295) was acquired from Abcam (Cambridge, MA, USA). The antibodies against alpha-smooth muscle actin (αSMA) were purchased from Abcam (ab49481) and GeneTex (GTX100034, San Antonio, TX, USA). All antibodies were used according to the manufacture’s recommendations. The HRP-conjugated secondary antibodies were obtained from Jackson ImmunoResearch Laboratories (West Grove, PA, USA).

### 2.4. Experimental Design

After acclimatization for at least one week, the animals were randomly recruited into two groups: (1) sham-operated rats (*n* = 10) and (2) UUO group (*n* = 20), which was subdivided into two groups corresponding to 14 and 28 days of obstruction (*n* = 10, respectively). First, the rats were anesthetized with 3% isoflurane using the Low-Flow Digital Anesthesia system for mice and rats SomnoSuite^®^ (Kent Scientific Corporation, Torrington, CT, USA). After the absence of a response to pain, determined by pedal withdrawal reflex, the procedure of the UUO model was performed. Briefly, an abdominal midline incision was performed under a stereoscopic microscope (Leica, Wetzlar, Germany). Next, the ureter was visualized and dissected, then the double ligation of the left ureter was performed, 2 cm below the kidney with 3–0 silk sutures. Once the muscle and skin were sutured, the rats were kept in regular cages with a temperature-controlled environment and given water and food *ad libitum*. At the end of each time point, the animals were euthanized under intraperitoneal anesthesia (sodium pentobarbital, 120 mg/100 g body weight). The sham animals were sacrificed at 28 days under the same conditions as the experimental groups. The kidneys and hearts were collected in cold phosphate-buffered saline solution for the corresponding assays and stored at −70 °C until use. The animals were subdivided for different determinations. Four animals per group were used for kidney histological analyses and Western blot determinations in renal and cardiac tissues, and six animals were used for pro-inflammatory cytokines content in blood serum and bioenergetics evaluation in isolated heart mitochondria.

### 2.5. Assessment of Tubular Damage and Fibrosis by Histological Analysis

Sagittal sections of the kidney were immersed in a 4% *p*-formaldehyde plus 1.5% glutaraldehyde solution, embedded in paraffin, sectioned at 4 μm in a rotating microtome Leica RM 2125RT (Leica Biosystems, Wetzlar, Germany). Sections were stained with hematoxylin-eosin (H&E) or Sirius red, and at least two independent fields from two different sections were analyzed using Carl Zeiss Primo Star Image Analyzer with an integrated Zeiss Axiocam ERc 5S camera (Leica Camera Inc., Wetzlar, Germany) [15].

### 2.6. Blood Serum Biochemistry

At the end of the experiments, approximately two milliliters of blood were obtained from sham and UUO rats (*n* = 3–4). Serum was recovered after centrifugation for 10 min at 3000× *g* at 4 °C for blood urea nitrogen (BUN) and creatinine analyses. The assay kits were acquired from SPINREACT (Girona, Spain) and were performed following the manufacturer’s instructions.

### 2.7. Evaluation of Cardiac Function by Echocardiography

Rats of different experimental groups (*n* = 9–10) were anesthetized with a low dose of sodium pentobarbital (19 mg/100 g body weight, intraperitoneally) for echocardiographic analysis using a SONOS 550 echocardiographer (Koninklijke Philips Electronics, Eindhoven, The Netherlands) with a 12 MHz transducer. Briefly, the parasternal short and long axes were analyzed by two-dimensional M-mode echocardiography in each animal. In addition, LV cavity and thickness were measured to calculate ejection fraction (EF) using the formula: %EF = Y + ((100 − Y) × 0.15)), where Y = (LVEDd^2^ − LVEDs^2^/LVEDd^2^) × 100 as well as fractional shortening (FS) by the formula: %FS = ((LVEDd − LVEDs/LVEDd) × 100), where LVEDd and LVEDs are the LV dimension at end-diastole and end-systole, respectively, according to our previous report [15]. At the end of the evaluation, the animals were allowed to recover for a few days before being euthanized.

### 2.8. Determination of Pro-Inflammatory Cytokines

The content of IL-1, IL-6, TNF-α was evaluated in serum by enzyme-linked immunosorbent assay (ELISA) (PeproTech, Rocky Hill, NJ, USA) according to Pavón et al. [16].

### 2.9. Tissue Preparation

Cardiac and renal tissues stored at −70 °C were ground to powder in a frozen mortar using liquid nitrogen. Then, the pulverized tissue was dissolved in cold radioimmunoprecipitation assay (RIPA) buffer containing 20 mM Tris-HCl, pH 7.5, 150 mM NaCl, 0.1% SDS, 0.5% sodium deoxycholate, and 1 mM PMSF supplemented with protease inhibitors (SigmaFast™, Sigma-Aldrich). The homogenates were centrifuged at 15,000× *g* for 10 min at 4 °C; the supernatants were transferred into new tubes and stored at −70 °C.

### 2.10. Cardiac Mitochondria Isolation

Cardiac mitochondria were isolated as previously described by differential centrifugation in 4 °C isolation buffer (225 mM D-mannitol, 75 mM sucrose, 1 mM EDTA, 5 mM HEPES, 0.1% FA-free BSA, pH 7.4) [17]. The final mitochondrial pellet was resuspended in 180 μL of BSA-free isolation buffer, and the total mitochondrial protein was measured according to Lowry et al. [18].

### 2.11. Mitochondrial Oxygen Consumption, Transmembranal Potential (∆Ψm), and ATP Synthase Activity

Evaluation of mitochondrial oxygen consumption was performed with a high-resolution respirometer (Oxygraph O2k, Oroboros, Innsbruck, Austria) at 37 °C. Isolated mitochondria (200 μg of total protein) were loaded into the chamber containing 2 mL of respiration buffer MiR05 (0.5 mM EGTA, 3 mM MgCl_2_, 60 mM K-lactobionate, 20 mM taurine, 10 mM KH_2_PO_4_, 20 mM HEPES, 110 mM sucrose, and 0.1% essentially FA-free BSA, pH 7.4). Electron transport was started by the addition of Complex I (CI)-linked substrates (5 mM pyruvate, 2 mM malate, and 10 mM glutamate) and Complex II (CII)-linked substrates (10 mM succinate plus 0.5 μM rotenone) [19]. Respiration in state 3 (S3) was achieved by adding 2.5 mM ADP and state 4 (S4) by adding 2.5 μM oligomycin. All parameters were corrected by residual respiration values obtained by adding 0.5 μM rotenone plus 2.5 μM antimycin A. The respiratory control (RC) was defined as the S3/S4 ratio, and oxidative phosphorylation (OxPhos)-associated respiration (P) was defined as S3−S4 [19,20]. All values were normalized by total protein content. On the other hand, the changes in ∆Ψm for the different respiratory states were measured as previously reported [21]. Briefly, the changes in safranin O fluorescence (2 μM) were used to determine the ∆Ψm. The respective substrates were added to stimulate CI or CII. ∆Ψm in S3 was obtained by addition of 2.5 mM ADP and in S4 by addition of 2.5 μM oligomycin; a total of 5 μM CCCP was added to dissipate the ∆Ψm completely and to correct by the non-specific interactions. Results were expressed as the changes in the measurable concentration of safranin O (∆μM of S) in S3 or S4 concerning CCCP decoupling, and the results were normalized per milligram of protein (∆μM of S/mg of protein). Finally, the ATP synthase activity was measured following the reduction in NADP^+^ at 340 nm using an enzyme-linked assay [22]. The absorbance measurements were performed at 37 °C using a Synergy BioTek microplate reader (BioTek Instruments, Winooski, VT, USA).

### 2.12. Activity of Antioxidant Enzymes in Mitochondria

Isolated mitochondria were used for the measurement of antioxidant enzyme activities as previously described [21]. Briefly, glutathione peroxidase (GPx) activity was measured by the disappearance of NADPH at 340 nm in a coupled reaction with glutathione reductase (GR). Next, catalase activity was measured based on the disappearance of hydrogen peroxide (30 mM) at 240 nm [23], and data were expressed as *k*/mg of protein (*k* is the first-order constant). Finally, GR activity was evaluated by measuring the disappearance of NADPH at 340 nm. All absorbance measurements were performed at 37 °C using a Synergy-Biotek microplate reader (Biotek Instruments, Winooski, VT, USA).

### 2.13. Immunoblot Analysis

Proteins (30 μg) of each sample were diluted 1:1 with Laemmli 2× concentrate sample buffer (Sigma-Aldrich) and denatured by boiling for 5 min. The membranes of polyvinylidene difluoride (PVDF) (Immobilon^®^-P, Millipore, Billerica, MA, USA) were incubated overnight at 4 °C with primary antibodies at the indicated dilutions: α-SMA (1:4000, GeneTex), α-SMA (1:2000, Abcam), FN (1:2000), and TnT (1:5000). Then, incubation with HRP-conjugated secondary antibodies (1:20,000) was realized for 1 h at room temperature with constant agitation. The immunoblotted proteins were visualized using an Immobilon™ Western Chemiluminescent HRP Substrate detection system (Millipore, Billerica, MA, USA). Each membrane was incubated with anti-GAPDH (1:1000) used as a loading control. All images were analyzed using ImageJ (NIH, Bethesda, MD, USA) and reported as relative expression.

### 2.14. Statistical Analysis

All data were expressed as mean ± standard error of the mean (SEM) and analyzed with GraphPad Prism version 8 (San Diego, CA, USA). Significance was set at *p* ≤ 0.05 using one-way analysis of variance (ANOVA) followed by a Tukey or Dunnett test.

## 3. Results

### 3.1. Development of the UUO Model

**Figure 1:**
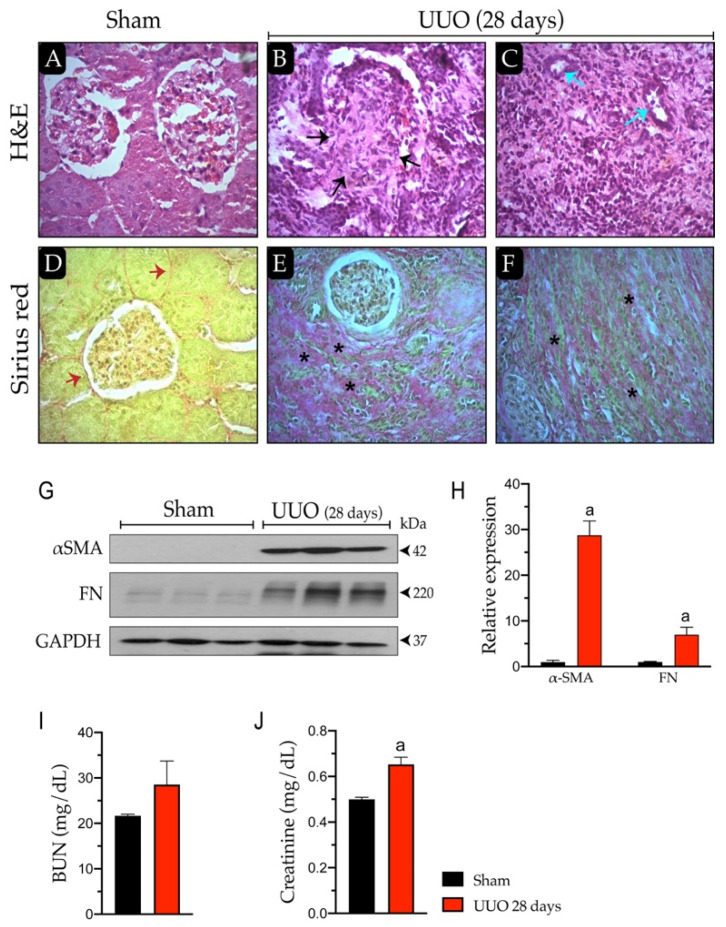
Characterization of the unilateral ureteral obstruction (UUO) model. Hematoxylin-eosin (H&E) staining: (**A**) sham and (**B**,**C**) 28-day UUO and Sirius red staining; (**D**) sham and (**E**,**F**) 28-day UUO analyzed by optical microscopy (scale bars: 40×). Glomerular sclerosis (black arrow); residual tubular structures (blue arrows), stroma (red arrows), and area of medulla positive Sirius red staining (black asterisks). (**G**) Immunodetection of proteins related to renal fibrosis. (**H**) Densitometric analysis of alpha-smooth muscle actin (αSMA) and fibronectin (FN). (**I**) Blood urea nitrogen (BUN) and (**J**) creatinine levels were measured in serum samples. Data represent mean ± SEM of 3–4 independent experiments. ^a^ *p* *≤* 0.05 vs. sham. GAPDH: Glyceraldehyde-3-phosphate dehydrogenase.

To confirm the development of the UUO model in rats, we evaluated fibrotic markers and the cellular architecture of the kidney. Figure 1A shows the glomerulus, Bowman’s capsule, and capillary network surrounded by mesangium and podocytes in the sham group. Animals with 28 days of obstruction had glomerular sclerosis, loss of capillaries, and fusion with Bowman’s capsule that continued towards the tubulointerstitial structures (black arrows); this sclerosis corresponds to 90% of the glomerular damage (Figure 1B). Moreover, fibrosis and reactive lymphocytes were observed in the tubulointerstitial zone and the residual tubular structures (Figure 1C, blue arrows). In Sirius red staining, only the positive area for stromal staining was visible (Figure 1D, red arrows).

On the other hand, we observed residual glomerulus with a clear area of tubulointerstitial fibrosis replacing the renal parenchymal tubule (Figure 1E, black asterisks) and Sirius red-positive areas in the medullary zone with fibrosis and loss of histological architecture (Figure 1F, black asterisks).

We also immunodetected proteins related to the fibrotic response in renal damage (Figure 1G). It was observed that αSMA increased (*p* ≤ 0.05) in the UUO group (up to 30-fold) compared to the sham group. Similarly, FN augmented (*p* ≤ 0.05) in the UUO animals by up to 7-fold versus sham (Figure 1H). We further characterized renal dysfunction by evaluating BUN and creatinine levels in blood serum. However, we only observed a significant increase in creatinine (*p* ≤ 0.05) in animals with 28 days of obstruction (0.65 ± 0.03 mg/dL) compared to the sham animals (0.49 ± 0.009 mg/dL) (Figure 1J); meanwhile, BUN was not modified (Figure 1I). With the above, we confirmed that the UUO model developed renal damage based on changes in the renal architecture.

### 3.2. Cardiac Function

**Figure 2:**
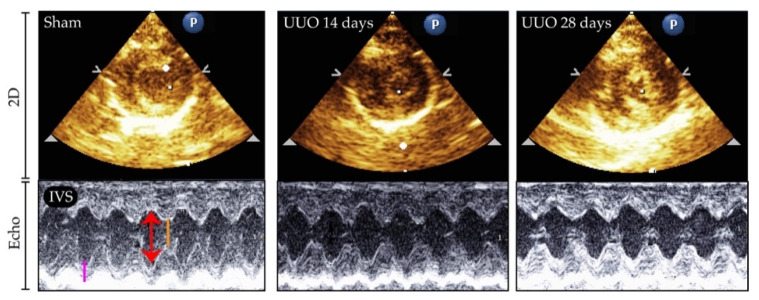
Representative two-dimension (2D) image and echocardiographic (Echo) analysis in the left ventricle (LV) of animals with unilateral ureteral obstruction (UUO) for 14 and 28 days. IVS: interventricular septum; LVEDd: LV dimension at end-diastole (red arrow); LVEDs: LV dimension at end-systole (orange line); LVPW: LV dimension at the posterior wall (pink line).

After characterization of the UUO model, we evaluated if kidney damage might change cardiac function at two different times of evolution. Therefore, another set of animals was subjected to ureteral obstruction and evaluated after 14 days. At both time frames (14 and 28 days), cardiac function was assessed by echocardiography (Figure 2).

Structural parameters such as the interventricular septum (IVS), left ventricular dimension at end-systole (LVEDs), and left ventricular at end-diastole (LVEDd) were unchanged at both 14 and 28 days of obstruction compared to sham (Table 1). Ejection fraction, which is the most accurate parameter to evaluated cardiac function, did not show changes either. Accordingly, the ratio of heart weight (HW) and lung weight (LW) to tibia length (TL) was similar in all groups (Table 1). In general, the renal damage generated after ureteral obstruction at 14 and 28 days did not promote heart dysfunction.

### 3.3. Proteins Related to Cardiac Damage and Inflammation

**Figure 3:**
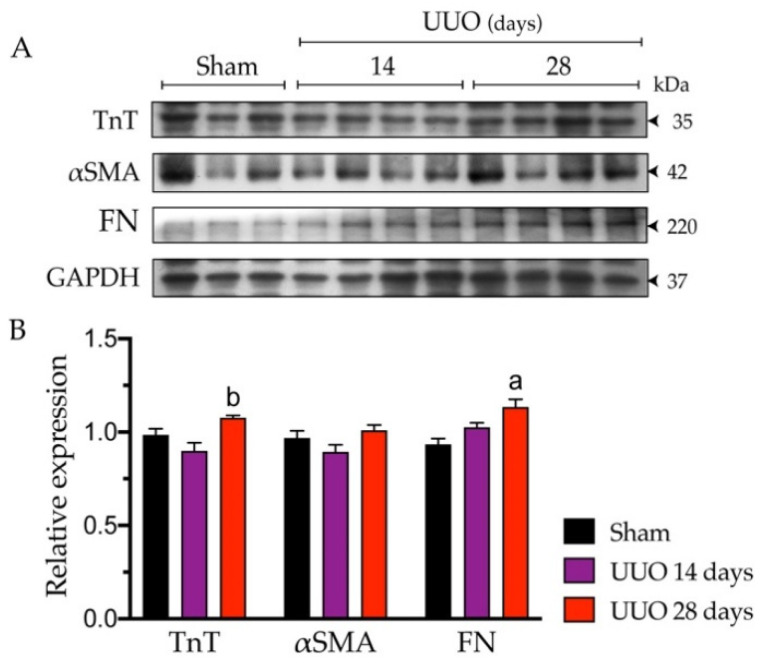
Measurements of proteins related to cardiac damage in animals with 14 and 28 days of unilateral ureteral obstruction (UUO). (**A**) Western blot image. (**B**) Quantitative immunoblot analysis. Data represent mean ± SEM of 3–4 independent experiments. ^a^ *p* ≤ 0.05 vs. sham; ^b^ *p* ≤ 0.05 vs. UUO 14 days. TnT: troponin T; αSMA: alpha-smooth muscle actin; FN: fibronectin; GAPDH: glyceraldehyde-3-phosphate dehydrogenase.

It has been documented that under certain circumstances, e.g., in aging, the heart can activate protective or compensatory mechanisms before developing cardiac dysfunction and, therefore, myocardial damage [15]. Under this premise, we performed immunodetection of TnT (a marker of cardiac damage) and fibrosis-related markers. As shown in Figure 3, TnT protein content slightly increased (*p* ≤ 0.05) in animals with 28 days of obstruction compared to those with 14 days. In contrast, the αSMA expression was unchanged, while FN increased by 15% in the animals with 28 days of obstruction versus sham. Supplementary Figure S1 shows the images of the untrimmed membranes of Western blots.

Moreover, we determined inflammatory markers by ELISA assay. Table 2 shows the inflammatory profile in the serum of sham and UUO animals with 14 and 28 days of obstruction. Both TNF-α (*p* ≤ 0.05) and IL-1 (*p* ≤ 0.05) increased at 28 days compared to the sham group, with no changes in IL-6. Furthermore, we evaluated renal function at 14 days and observed that neither BUN nor creatinine was modified at this time compared to animals with 28 days of evolution.

### 3.4. Mitochondrial Respiration

**Figure 4:**
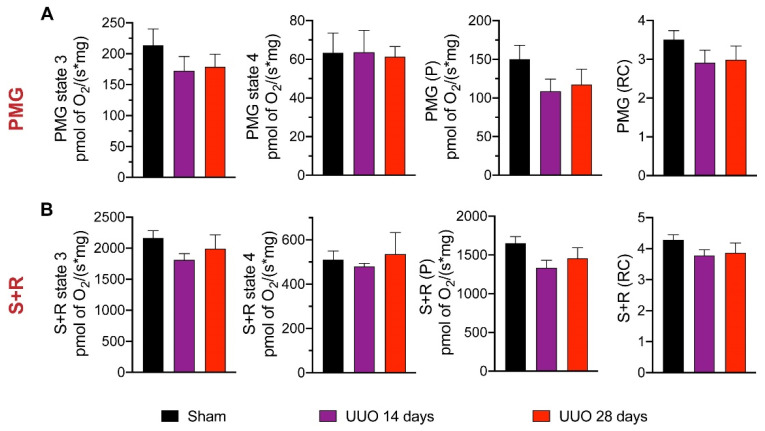
Mitochondrial respiration parameters in cardiac tissue of rats with unilateral ureteral obstruction (UUO) for 14 and 28 days. Respiratory parameter-related data for (**A**) CI-linked respiration and (**B**) CII-linked respiration: state 3 (S3), oligomycin-induced state 4 (S4), oxidative phosphorylation (OxPhos)-associated respiration (P), and respiratory control (RC). Data represent mean ± SEM of 5–6 independent experiments. PMG: pyruvate-malate-glutamate; S+R: succinate plus rotenone; CI: Complex I; CII: Complex II.

As mitochondrial damage was associated with kidney injury in several models [10,11,17,19], we used a high-resolution respiration protocol to assess CI- and CII-linked respiration in isolated cardiac mitochondria from UUO rats subjected to the 14- and 28-day protocol. Using pyruvate-malate-glutamate (PMG, Figure 4A), S3, S4, P, and RC were unchanged. Analogously, using succinate + rotenone (S + R) to evaluate CII-linked respiration did not show significant differences between groups (Figure 4B).

**Figure 5:**
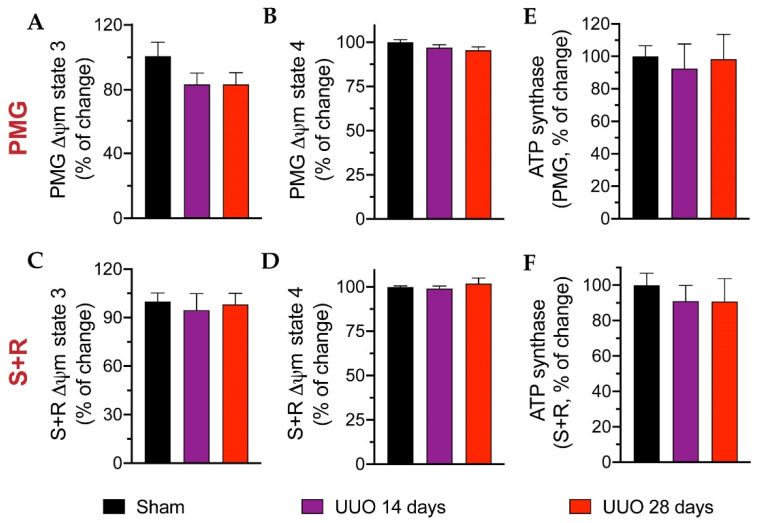
Mitochondrial integrity and ATP synthase activity in cardiac mitochondria isolated from unilateral ureteral obstruction (UUO) animals for 14 and 28 days. Data related to transmembranal potential (∆Ψm) using substrates for Complex I (CI) at (**A**) state 3 (S3) and (**B**) state 4 (S4), and for Complex II (CII) at (**C**) S3 and (**D**) S4. ATP synthase activity using (**E**) pyruvate-malate-glutamate (PMG) and (**F**) succinate + rotenone (S + R) substrates, respectively. Data represent the mean ± SEM of 4–5 independent experiments.

**Figure 6:**
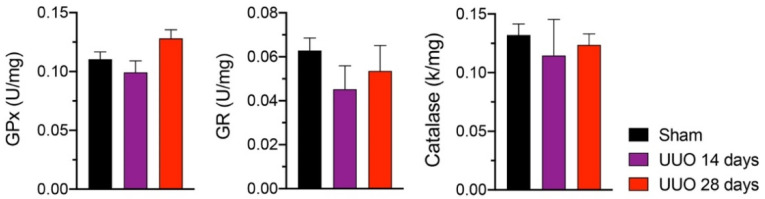
Antioxidant enzyme activity in isolated cardiac mitochondria from rats with unilateral ureteral obstruction (UUO) for 14 and 28 days. Data represent mean ± SEM of 4–5 independent experiments. GPx: glutathione peroxidase; GR: glutathione reductase.

Likewise, we measured the ∆Ψm using the PMG substrate at S3 (Figure 5A) and S4 (Figure 5B) as well as using the S + R substrate at S3 (Figure 5C) and S4 (Figure 5D); however, no significant changes were apparent in either group. Finally, ATP synthase activity was measured, but again no differences were found (Figure 5E,F). Although mitochondrial integrity and function were not modified, we analyzed the activity of three antioxidant enzymes: GPx, GR, and catalase (Figure 6). We observed a modest increase, albeit not statistically significant, in GPx activity in animals with 28 days of obstruction compared to rats from sham and 14 days. The activities of GR and catalase tended to decrease in animals with obstruction without reaching significance.

## 4. Discussion

CKD and end-stage renal disease prevalence have progressively increased in developed countries due to rising risk factors such as diabetes, obesity, and hypertension [24,25]. Additionally, cardiac dysfunction has been widely reported to be highly prevalent among CKD patients, especially those on dialysis [26,27]. Animals with severe CKD induced by 5/6 nephrectomy showed diastolic dysfunction and ventricular hypertrophy at four weeks and progressive cardiac fibrosis and heart failure after eight weeks of kidney injury [28]. In this regard, the term coined as a cardio-renal syndrome (CRS) derives from recognizing the bidirectional links between cardiac and renal functions and the understanding that the dysfunction of one organ affects the other [29]. However, the underlying pathophysiology of CRS is complex and not yet clearly described [30].

Animal models of 5/6 nephrectomy or bilateral ischemia-reperfusion injury have served to delineate the primary mechanism underpinning the different subtypes of CRS [31,32]. Thus far, in the UUO-induced CKD model, neither the damaged relationship between the two organs nor in what time frame pathological cardiac remodeling occurs in the presence of ureteral obstruction-induced renal dysfunction in rats has been reported. However, the UUO model is essential in the clinical, as acute and chronic urinary obstructions due to benign prostatic hypertrophy, renal calculi, or other urinary retention are frequently observed. Therefore, the elucidation of possible damage to other tissues would help to improve the long-term management of patients.

Recent work reports the presence of mild to moderate injury in the UUO model, possibly due to the compensatory function of the entire kidney [9]. Our data revealed that renal damage was generated after ureteral obstruction at 14 and 28 days (Figure 1); however, it did not concur with changes in cardiac function (Table 1). In contrast, other authors indicate that pathological cardiac remodeling occurs three weeks after UUO in mice [9]. Nevertheless, it is essential to emphasize that the experimental design of the report by Ham et al. [9] does not include the sham group but control animals. Therefore, the pathological cardiac remodeling occurring three weeks after UUO in these mice cannot be evenly compared, and the rest of the parameters evaluated in such a study do not necessarily reflect the effects of the obstruction.

It has been reported that up to 2 months of obstruction progression are required to detect inflammation, fibrosis, and changes in cardiac function in mice [6]. In this regard, even if in our experimental model 28 days of obstruction were sufficient to appreciate significant changes in IL-1β and TNF-α (Table 2) along with a modest increase in FN and TnT (Figure 3), it was not enough for the establishment of cardiac dysfunction (Table 1). It is essential to recognize and understand that the heart activates underlying molecular mechanisms before cardiac dysfunction develops [15], which may delay the onset and progression of cardiovascular complications in patients with advanced CKD [33,34,35].

Mitochondrial function is of crucial importance in the heart due to high energy demand. Mitochondria are also involved in metabolic processes such as steroid and heme biosynthesis, calcium homeostasis, programmed cell death, innate immunity, and communication with other organelles to maintain cellular homeostasis [36,37,38]. Numerous cardiovascular diseases are associated with mitochondrial dysfunction [39,40], e.g., cardiac hypertrophy, myocardial ischemia/reperfusion, and heart failure [40,41]. In our study, both mitochondrial integrity and function were not altered (Figure 4 and Figure 5 and Table 1), despite apparent renal damage (Figure 1). Recently, Bianco et al. [13] reported that after 14 days of UUO progression in mice, the kidney exhibited altered mitochondrial function and impaired redox homeostasis; however, data related to mitochondrial bioenergetics of the heart were not evaluated in their report. Finally, although global renal dysfunction may be variable and difficult to predict in this model, one possible reason is related to the compensatory function exerted by the contralateral kidney. In this model, it was described that the contralateral kidney began to grow 20 to 30 days after ureteral ligation to compensate for the unilateral loss of renal function. This change reflects a structural adaptation due to the increased glomerular filtration rate, causing compensatory hypertrophy [2,13]. Even the evolution time in our model was relatively short; the UUO model could help detect the early impact of CKD on the development of possible cardiac remodeling before clinical symptoms manifest.

UUO is an essential model for the study of mechanisms of renal fibrosis and evaluating the impact of potential therapeutic approaches to ameliorate renal disease. Many quantifiable pathophysiological events occur for one week of UUO. Acute and chronic urinary obstruction from benign prostate hypertrophy, kidney stones, or various other urinary retentions is commonly seen in the clinical setting. As well as a significant cause of renal failure in children and infants is congenital obstructive nephropathy [4,5]. Some of the animal model findings have been compared with observations made in patients with obstructive nephropathy, and most of the evidence suggests that the rodent model of UUO is reflective of human renal disease processes.

The main limitation of our study was the time of progression of the UUO model in rats; however, this fact could be a double-edged sword. It is possible that if we were to prolong the model to 2 months of evolution, changes in cardiac function and a possible compensatory mechanism after renal damage could be observed (offering a CRS model). Nevertheless, the mortality rate at 28 days was very high, complicating the analysis of future experimental determinations. On the other hand, an attractive perspective to examine in our model was the role of mitochondrial dynamics. Although we assessed mitochondrial function, we do not know whether proteins involved in the process of mitochondrial fusion/fission, mitophagy or mitochondrial biogenesis could have been altered from early times (14 days) and stabilized at 28 days, which could explain (at least partially) why mitochondrial function and integrity was not modified. Thus, these points would offer us a complete scenario of the mitochondria in the UUO model in rats.

## 5. Conclusions

**Figure 7:**
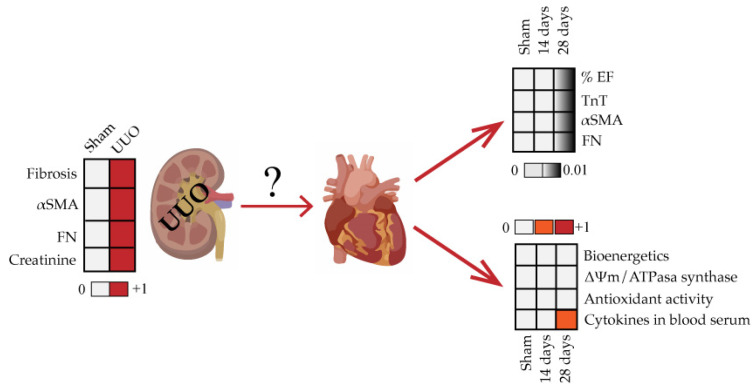
Integrative scheme. In summary, the results of our study show that notwithstanding the establishment of the UUO model in rats (determined by fibrosis markers), cardiac function (assessed by %EF, TnT, αSMA, and FN), and mitochondrial integrity in these animals were not affected. However, the animals showed an increase in inflammation up to 28 days measured in blood serum. UUO: unilateral ureteral obstruction; α–SMA: alpha-smooth muscle actin; FN: fibronectin; %EF: percentage of ejection fraction; TnT: troponin T; ∆Ψm: transmembrane potential.

Our data indicate no pathological cardiac remodeling in CKD induced by the UUO model in the rat after 28 days of evolution; even though inflammation was present during the evolution of this condition (Figure 7).

## Figures and Tables

**Table 1:** Cardiac structural and functional parameters in rats subjected to unilateral ureteral obstruction (UUO) for 14 and 28 days. Data are the mean ± SEM of 9–10 different animals in each experimental group. IVS: interventricular septum; LVEDd: LV dimension at end-diastole; LVEDs: LV dimension at end-systole; LVPW: LV dimension posterior wall, EF: ejection fraction; FS: fractional shortening; HR: heart rate; bpm: beats per minute.

		UUO (Days)	
Parameter	Sham	14	28
IVS (mm)	0.20 ± 0.003	0.20 ± 0.002	0.198 ± 0.002
LVEDd (mm)	5.37 ± 0.07	5.25 ± 0.05	5.46 ± 0.05
LVEDs (mm)	2.46 ± 0.04	2.34 ± 0.05	2.28 ± 0.06
LVPW (mm)	0.19 ± 0.003	0.18 ± 0.002	0.19 ± 0.003
EF (%)	82.1 ± 0.44	83.7 ± 0.5	85.1 ± 0.6
FS (%)	54.22 ± 0.57	55.7 ± 0.7	58.5 ± 0.9
HR (bpm)	413.79 ± 6.7	433.1 ± 6.7	417.0 ± 7.9
Body weight (BW, g)	373.3 ± 4.2	350.7 ± 4.7	360.0 ± 3.6
Heart weight (HW, g)	1.41 ± 0.05	1.19 ± 0.02	1.31 ± 0.03
Lung weight (LW, g)	2.21 ± 0.08	1.92 ± 0.03	1.94 ± 0.05
Tibial length (TL, cm)	5.3 ± 0.03	5.3 ± 0.02	5.4 ± 0.02
HW/TL (g/cm)	0.25 ± 0.01	0.22 ± 0.005	0.24 ± 0.01
LW/TL (g/cm)	0.36 ± 0.01	0.36 ± 0.006	0.36 ± 0.01
HW/BW (g/kg)	3.57 ± 0.11	3.40 ± 0.06	3.64 ± 0.08
LW/BW (g/kg)	5.20 ± 0.20	5.54 ± 0.12	5.38 ± 0.12
Functional parameters	(*n* = 9)	(*n* = 10)	(*n* = 10)

**Table 2:** Pro-inflammatory cytokine content and enzymes related to renal function in blood serum. Pro-inflammatory cytokines content in blood serum obtained from sham and unilateral ureteral obstruction (UUO) rats at 14 and 28 days by enzyme-linked immunosorbent assay (ELISA), as well as the creatinine and blood urea nitrogen (BUN) levels, were determined. Data are the mean ± SEM of 5–6 different animals in each experimental group ^a^ *p* ≤ 0.05 vs. sham; ^b^ *p* ≤ 0.05 vs. UUO 14 days; ^c^ *p* ≤ 0.05 vs. UUO 14 days. IL-6: interleukin-1; IL-6: interleukin-6; TNF-α: tumor necrosis factor alpha.

		UUO (Days)	
	Sham	14	28
IL-1 (pg/mg)	10.56 ± 0.5	10.67 ± 0.88	21.14 ± 1.9 ^a,b^
IL-6 (pg/mg)	27.73 ± 10.76	11.98 ± 1.15	36.66 ± 13.27
TNF-α (pg/mg)	9.41 ± 0.41	8.77 ± 1.14	14.79 ± 2.16 ^c^
BUN (mg/dL)	21.67 ± 0.33	23.25 ± 2.05	28.50 ± 5.18
Creatinine (mg/dL)	0.49 ± 0.009	0.61 ± 0.02	0.65 ± 0.03 ^a^

## Data Availability

Not applicable.

## Supplementary Materials

The following are available online at https://www.mdpi.com/article/10.3390/biology10070671/s1, Figure S1: Untrimmed membranes of Western blots.

## References

[1] Kaeidi A., Sahamsizadeh A., Allahtavakoli M., Fatemi I., Rahmani M., Hakimizadeh E., Hassanshahi J. (2020). The effect of oleuropein on unilateral ureteral obstruction induced-kidney injury in rats: The role of oxidative stress, inflammation and apoptosis. Mol. Biol. Rep..

[2] Chevalier R.L., Forbes M.S., Thornhill B.A. (2009). Ureteral obstruction as a model of renal interstitial fibrosis and obstructive nephropathy. Kidney Int..

[3] Martínez-Klimova E., Aparicio-Trejo O.E., Tapia E., Pedraza-Chaverri J. (2019). Unilateral Ureteral Obstruction as a Model to Investigate Fibrosis-Attenuating Treatments. Biomolecules.

[4] Klahr S., Morrissey J. (2002). Obstructive nephropathy and renal fibrosis. Am. J. Physiol. Renal Physiol..

[5] Manucha W. (2007). Biochemical-molecular markers in unilateral ureteral obstruction. Biocell.

[6] Prud’homme M., Coutrot M., Michel T., Boutin L., Genest M., Poirier F., Launay J.M., Kane B., Kinugasa S., Prakoura N. (2019). Acute Kidney Injury Induces Remote Cardiac Damage and Dysfunction Through the Galectin-3 Pathway. JACC Basic Transl. Sci..

[7] Wen Y., Lu X., Ren J., Privratsky J.R., Yang B., Rudemiller N.P., Zhang J., Griffiths R., Jain M.K., Nedospasov S.A. (2019). KLF4 in Macrophages Attenuates TNF*α*-Mediated Kidney Injury and Fibrosis. J. Am. Soc. Nephrol..

[8] Bhargava P., Schnellmann R.G. (2017). Mitochondrial energetics in the kidney. Nat. Rev. Nephrol..

[9] Ham O., Jin W., Lei L., Huang H.H., Tsuji K., Huang M., Roh J., Rosenzweig A., Lu H.A.J. (2018). Pathological cardiac remodeling occurs early in CKD mice from unilateral urinary obstruction, and is attenuated by Enalapril. Sci. Rep..

[10] Correa F., Buelna-Chontal M., Hernández-Reséndiz S., García-Niño W.R., Roldán F.J., Soto V., Silva-Palacios A., Amador A., Pedraza-Chaverrí J., Tapia E. (2013). Curcumin maintains cardiac and mitochondrial function in chronic kidney disease. Free Radic. Biol. Med..

[11] Rojas-Morales P., Tapia E., León-Contreras J.C., González-Reyes S., Jiménez-Osorio A.S., Trujillo J., Pavón N., Granados-Pineda J., Hernández-Pando R., Sánchez-Lozada L.G. (2019). Mechanisms of Fasting-Mediated Protection against Renal Injury and Fibrosis Development after Ischemic Acute Kidney Injury. Biomolecules.

[12] Martínez-Klimova E., Aparicio-Trejo O.E., Gómez-Sierra T., Jiménez-Uribe A.P., Bellido B., Pedraza-Chaverri J. (2020). Mitochondrial dysfunction and endoplasmic reticulum stress in the promotion of fibrosis in obstructive nephropathy induced by unilateral ureteral obstruction. Biofactors.

[13] Bianco M., Lopes J.A., Beiral H.J.V., Filho J.D.D., Frankenfeld S.P., Fortunato R.S., Gattass C.R., Vieyra A., Takiya C.M. (2019). The contralateral kidney presents with impaired mitochondrial functions and disrupted redox homeostasis after 14 days of unilateral ureteral obstruction in mice. PLoS ONE.

[14] Kilkenny C., Browne W.J., Cuthill I.C., Emerson M., Altman D.G. (2010). Improving bioscience research reporting: The ARRIVE guidelines for reporting animal research. PLoS Biol..

[15] Silva-Palacios A., Ostolga-Chavarría M., Buelna-Chontal M., Garibay C., Hernández-Reséndiz S., Roldán F.J., Flores P.L., Luna-López A., Königsberg M., Zazueta C. (2017). 3-NP-induced Huntington’s-like disease impairs Nrf2 activation without loss of cardiac function in aged rats. Exp. Gerontol..

[16] Pavón N., Aranda A., García N., Hernández-Esquivel L., Chávez E. (2009). In hyperthyroid rats octylguanidine protects the heart from reperfusion damage. Endocrine.

[17] Aparicio-Trejo O.E., Avila-Rojas S.H., Tapia E., Rojas-Morales P., León-Contreras J.C., Martínez-Klimova E., Hernández-Pando R., Sánchez-Lozada L.G., Pedraza-Chaverri J. (2020). Chronic impairment of mitochondrial bioenergetics and β-oxidation promotes experimental AKI-to-CKD transition induced by folic acid. Free Radic. Biol. Med..

[18] Lowry O.H., Rosebrough N.J., Farra A.L., Randall R.J. (1951). Protein measurement with the Folin phenol reagent. J. Biol. Chem..

[19] Aparicio-Trejo O.E., Rojas-Morales P., Avila-Rojas S.H., León-Contreras J.C., Hernández-Pando R., Jiménez-Uribe A.P., Prieto-Carrasco R., Sánchez-Lozada L.G., Pedraza-Chaverri J., Tapia E. (2020). Temporal Alterations in Mitochondrial β-Oxidation and Oxidative Stress Aggravate Chronic Kidney Disease Development in 5/6 Nephrectomy Induced Renal Damage. Int. J. Mol. Sci..

[20] Ojuka E., Andrew B., Bezuidenhout N., George S., Maarman G., Madlala H.P., Mendham A., Osiki P.O. (2016). Measurement of β-oxidation capacity of biological samples by respirometry: A review of principles and substrates. Am. J. Physiol. Endocrinol. Metab..

[21] Aparicio-Trejo O.E., Reyes-Fermín L.M., Briones-Herrera A., Tapia E., León-Contreras J.C., Hernández-Pando R., Sánchez-Lozada L.G., Pedraza-Chaverri J. (2019). Protective effects of N-acetyl-cysteine in mitochondria bioenergetics, oxidative stress, dynamics and S-glutathionylation alterations in acute kidney damage induced by folic acid. Free Radic. Biol. Med..

[22] Aparicio-Trejo O.E., Tapia E., Molina-Jijón E., Medina-Campos O.N., Macías-Ruvalcaba N.A., León-Contreras J.C., Hernández-Pando R., García-Arroyo F.E., Cristóbal M., Sánchez-Lozada L.G. (2017). Curcumin prevents mitochondrial dynamics disturbances in early 5/6 nephrectomy: Relation to oxidative stress and mitochondrial bioenergetics. Biofactors.

[23] Aebi H. (1984). Catalase in vitro. Methods Enzymol..

[24] Gajjala P.R., Sanati M., Jankowski J. (2015). Cellular and Molecular Mechanisms of Chronic Kidney Disease with Diabetes Mellitus and Cardiovascular Diseases as Its Comorbidities. Front. Immunol..

[25] Liu Y.W., Su C.T., Song E.J., Tsai W.C., Li Y.H., Tsai L.M., Chen J.H., Sung J.M. (2015). The role of echocardiographic study in patients with chronic kidney disease. J. Formos. Med. Assoc..

[26] Mitsnefes M.M. (2012). Cardiovascular disease in children with chronic kidney disease. J. Am. Soc. Nephrol..

[27] Cai Q., Mukku V.K., Ahmad M. (2013). Coronary artery disease in patients with chronic kidney disease: A clinical update. Curr. Cardiol. Rev..

[28] Winterberg P.D., Jiang R., Maxwell J.T., Wang B., Wagner M.B. (2016). Myocardial dysfunction occurs prior to changes in ventricular geometry in mice with chronic kidney disease (CKD). Physiol. Rep..

[29] McCullough P.A., Kellum J.A., Haase M., Müller C., Damman K., Murray P.T., Cruz D., House A.A., Schmidt-Ott K.M., Vescovo G. (2013). Pathophysiology of the cardiorenal syndromes: Executive summary from the eleventh consensus conference of the Acute Dialysis Quality Initiative (ADQI). Contrib. Nephrol..

[30] Das S., Aiba T., Rosenberg M., Hessler K., Xiao C., Quintero P.A., Ottaviano F.G., Knight A.C., Graham E.L., Boström P. (2012). Pathological role of serum- and glucocorticoid-regulated kinase 1 in adverse ventricular remodeling. Circulation.

[31] Bongartz L.G., Braam B., Gaillard C.A., Cramer M.J., Goldschmeding R., Verhaar M.C., Doevendans P.A., Joles J.A. (2012). Target organ cross talk in cardiorenal syndrome: Animal models. Am. J. Physiol. Renal Physiol..

[32] Hewitson T.D., Holt S.G., Smith E.R. (2015). Animal Models to Study Links between Cardiovascular Disease and Renal Failure and Their Relevance to Human Pathology. Front. Immunol..

[33] Yin J., Lu Z., Wang F., Jiang Z., Lu L., Miao N., Wang N. (2016). Renalase attenuates hypertension, renal injury and cardiac remodelling in rats with subtotal nephrectomy. J. Cell. Mol. Med..

[34] Husain-Syed F., McCullough P.A., Birk H.W., Renker M., Brocca A., Seeger W., Ronco C. (2015). Cardio-Pulmonary-Renal Interactions: A Multidisciplinary Approach. J. Am. Coll. Cardiol..

[35] Rangaswami J., Bhalla V., Blair J.E.A., Chang T.I., Costa S., Lentine K.L., Lerma E.V., Mezue K., Molitch M., Mullens W. (2019). American Heart Association Council on the Kidney in Cardiovascular Disease and Council on Clinical Cardiology. Cardiorenal Syndrome: Classification, Pathophysiology, Diagnosis, and Treatment Strategies: A Scientific Statement From the American Heart Association. Circulation.

[36] Saada A. (2011). The use of individual patient’s fibroblasts in the search for personalized treatment of nuclear encoded OXPHOS diseases. Mol. Genet. Metab..

[37] Saada A. (2014). Mitochondria: Mitochondrial OXPHOS (dys) function ex vivo–the use of primary fibroblasts. Int. J. Biochem. Cell. Biol..

[38] Silva-Palacios A., Zazueta C., Pedraza-Chaverri J. (2020). ER membranes associated with mitochondria: Possible therapeutic targets in heart-associated diseases. Pharmacol. Res..

[39] Dominic E.A., Ramezani A., Anker S.D., Verma M., Mehta N., Rao M. (2014). Mitochondrial cytopathies and cardiovascular disease. Heart.

[40] Ide T., Tsutsui H., Hayashidani S., Kang D., Suematsu N., Nakamura K., Utsumi H., Hamasaki N., Takeshita A. (2001). Mitochondrial DNA damage and dysfunction associated with oxidative stress in failing hearts after myocardial infarction. Circ. Res..

[41] Disatnik M.H., Ferreira J.C., Campos J.C., Gomes K.S., Dourado P.M., Qi X., Mochly-Rosen D. (2013). Acute inhibition of excessive mitochondrial fission after myocardial infarction prevents long-term cardiac dysfunction. J. Am. Heart Assoc..

