# Supplementary material for: Unilateral Ureteral Obstruction for 28 Days in Rats Is Not Associated with Changes in Cardiac Function or Alterations in Mitochondrial Function
Source: Biology (Basel). 2021 Jul 16;10(7):671. doi: 10.3390/biology10070671 (PMC8301354; doi:10.3390/biology10070671)

Uncropped blots: kidney markers

These immunoblots were used for the preparation of **Fig. 1G**

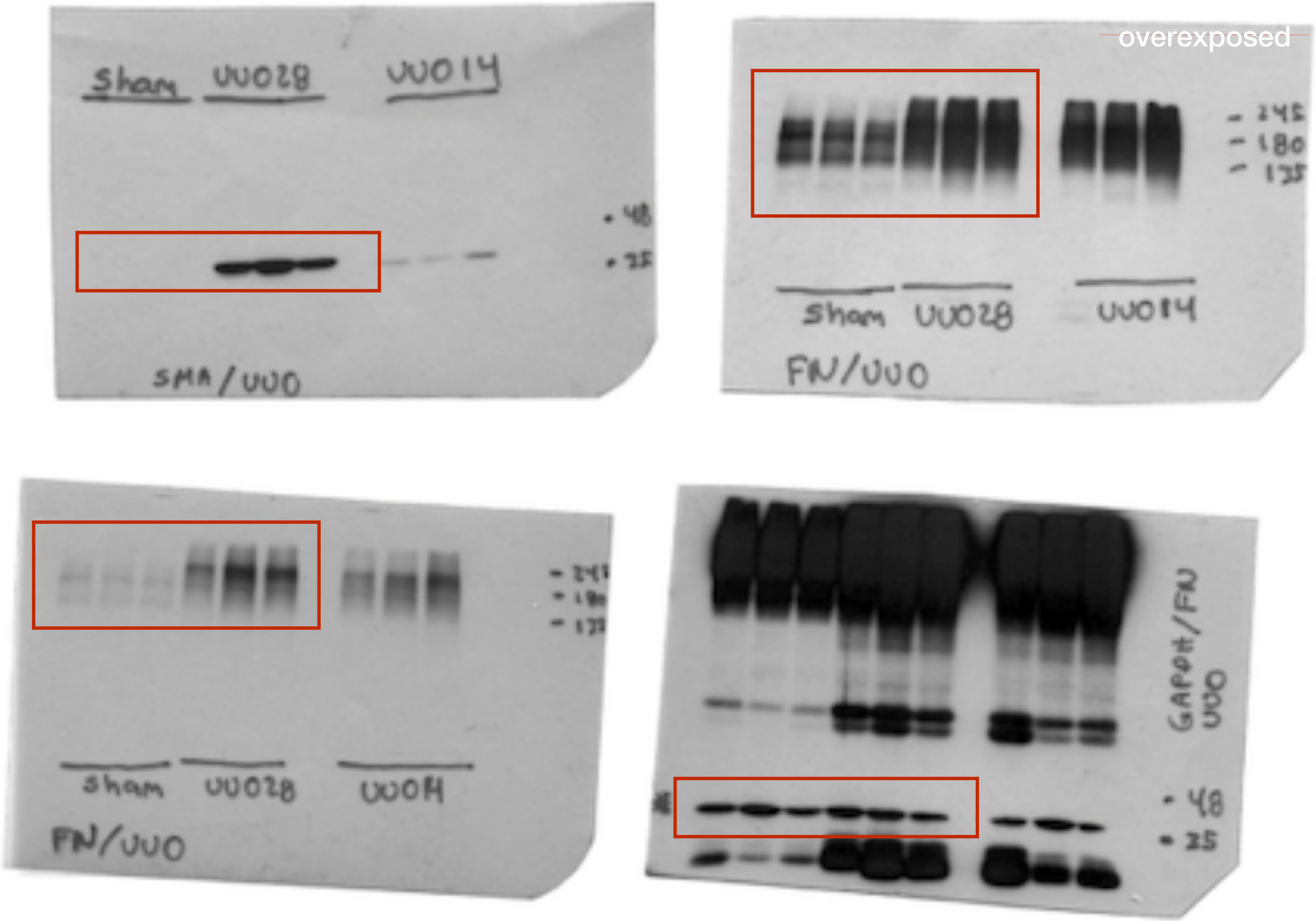

Uncropped blots: heart markers

These immunoblots were used for the preparation of **Fig. 3A**

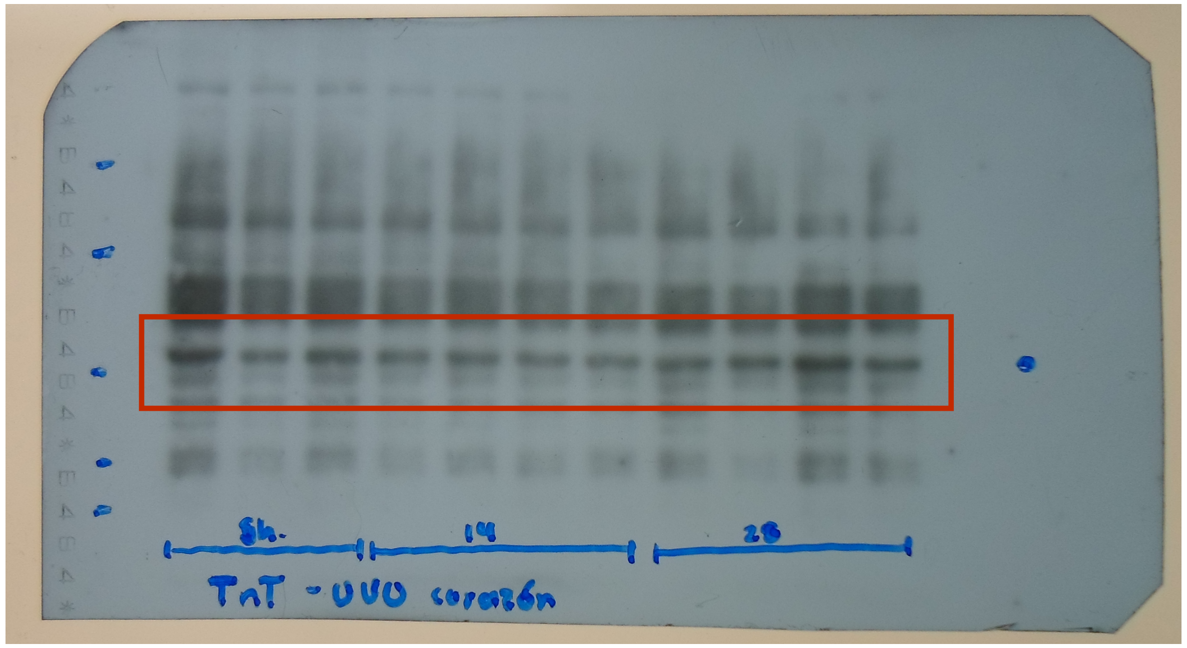

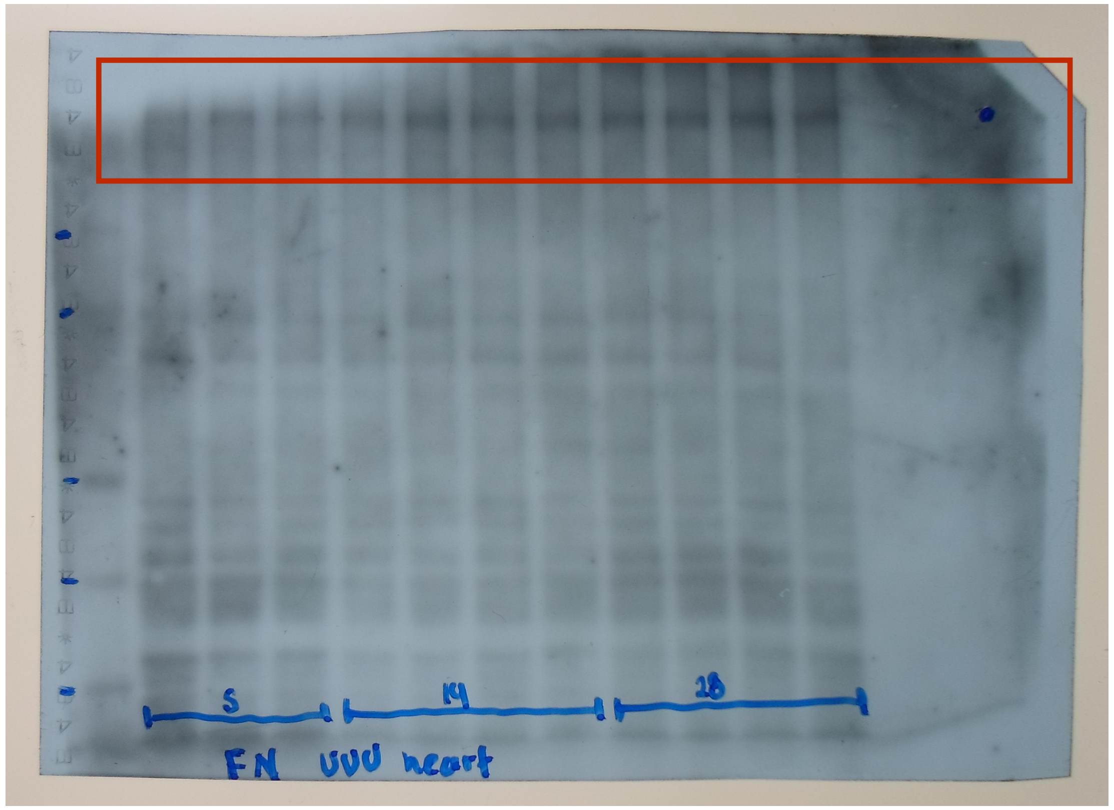

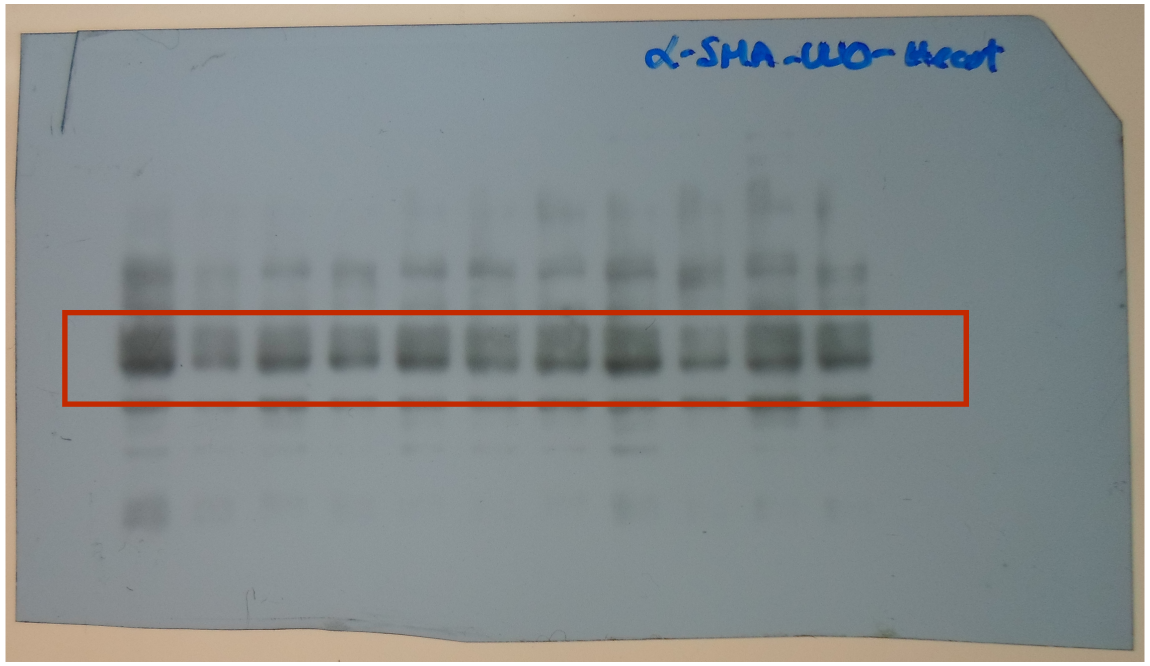

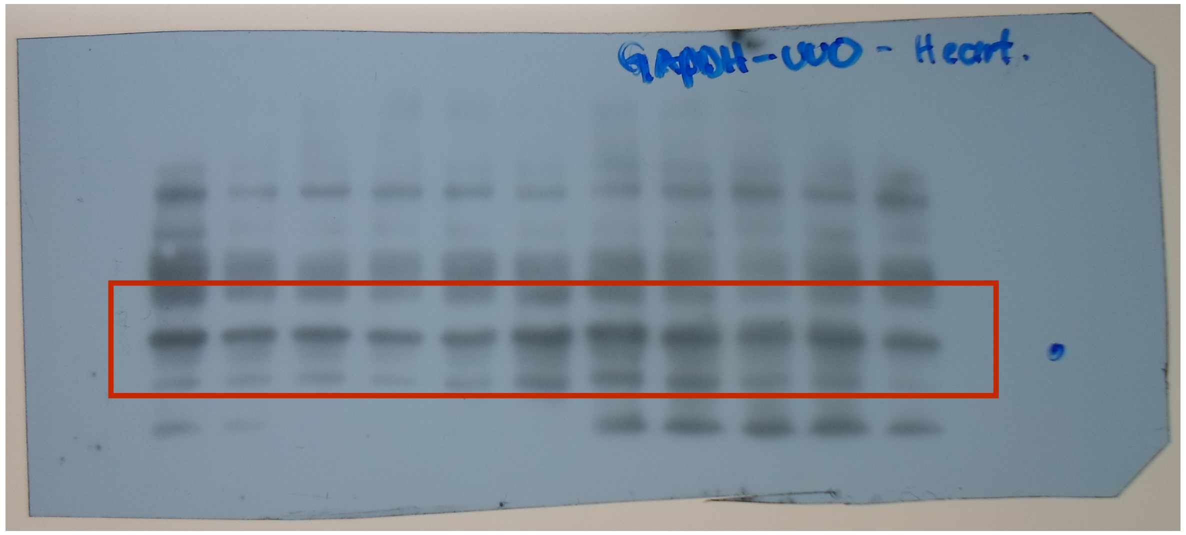

Supplement: Supplementary file 1 [file biology-10-00671-s001.zip › biology-1274940-supplementary.pdf]
